# Chromosome-Level Genome Assembly and Multi-Omics Dataset Provide Insights into Isoflavone and Puerarin Biosynthesis in *Pueraria lobata* (Wild.) Ohwi

**DOI:** 10.3390/biom12121731

**Published:** 2022-11-22

**Authors:** Hua Cheng, Xiaohua Huang, Shuai Wu, Shiyan Wang, Shen Rao, Li Li, Shuiyuan Cheng, Linling Li

**Affiliations:** 1 School of Modern Industry for Selenium Science and Engineering, Wuhan Polytechnic University, Wuhan 430048, China; 2 National R&D Center for Se-rich Agricultural Products Processing, Wuhan Polytechnic University, Wuhan 430023, China; 3 College of Biology and Agricultural Resources, Huanggang Normal University, Huanggang 438000, China

**Keywords:** genome assembly, positive selection analysis, *P. lobata* Ohwi, puerarin biosynthesis, regulatory network

## Abstract

*Pueraria lobata* (wild.) Ohwi is a leguminous plant and one of the traditional Chinese herbal medicines. Its puerarin extract is widely used in the pharmaceutical industry. This study reported a chromosome-level genome assembly for *P. lobata* and its characteristics. The genome size was ~939.2 Mb, with a contig N50 of 29.51 Mbp. Approximately 97.82% of the assembled sequences were represented by 11 pseudochromosomes. We identified that the repetitive sequences accounted for 63.50% of the *P. lobata* genome. A total of 33,171 coding genes were predicted, of which 97.34% could predict the function. Compared with other species, *P. lobata* had 757 species-specific gene families, including 1874 genes. The genome evolution analysis revealed that *P. lobata* was most closely related to *Glycine max* and underwent two whole-genome duplication (WGD) events. One was in a gamma event shared by the core dicotyledons at around 65 million years ago, and another was in the common ancestor shared by legume species at around 25 million years ago. The collinearity analysis showed that 61.45% of the genes (54,579 gene pairs) in *G. max* and *P. lobata* had collinearity. In this study, six unique *PlUGT43* homologous genes were retrieved from the genome of *P. lobata*, and no 2-hydroxyisoflavanone 8-C-glucoside was found in the metabolites. This also revealed that the puerarin synthesis was mainly from the glycation of daidzein. The combined transcriptome and metabolome analysis suggested that two *bHLHs*, six *MYBs* and four *WRKYs* were involved in the expression regulation of puerarin synthesis structural genes. The genetic information obtained in this study provided novel insights into the biological evolution of *P. lobata* and leguminous species, and it laid the foundation for further exploring the regulatory mechanism of puerarin synthesis.

## 1. Introduction

**Figure 1:**
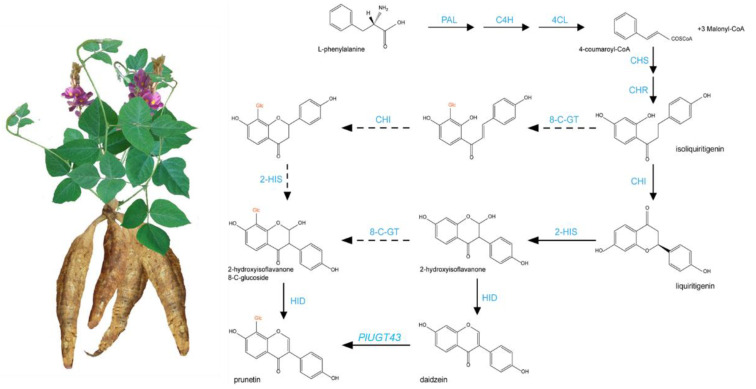
Basic pathway of puerarin biosynthesis and metabolism. Solid arrows indicate confirmed metabolic pathways, and dashed arrows indicate unconfirmed pathways.

*Pueraria lobata* (wild.) Ohwi (hereinafter abbreviated as *P. lobata*) is an important medicinal and edible plant, mainly distributed in East Asian countries, including China [1]. In traditional Chinese medicine, the root of *P. lobata* is the main medicinal component, also known as kudzu. A *Chinese Pharmacopeia* dating back to 200 B.C. mentions the use of the roots of kudzu and their use in various treatments [2]. Kudzu has long been used to treat fever, toxicosis, indigestion, alcoholism and other illnesses in the *Chinese Pharmacopoeia* [3]. The roots of *P. lobata* are rich resources of natural product isoflavonoids, including genistein, formononetin, daidzein and puerarin (also called daidzein 8-C-glycoside). Among these isoflavonoids, the main bioactive components are puerarin and daidzein [4,5]. Modern pharmacological studies have shown that these two isoflavones protect the cardiovascular system, exert an anti-inflammatory effect and reduce the blood alcohol levels [6,7]. Puerarin is considered to be the main active isoflavone of *P. lobata* and *P. thomsonii*. It has biological activities against cardiovascular disease, and vascular hypertension and improves insulin sensitivity [4,8,9]. Isoflavone synthesis is a branch of the flavonoid pathway. Puerarin is biosynthesized via the phenylpropanoid pathway by the hydroxylation of liquiritigenin at the C-2 position to yield its isoflavonoid skeleton [10] (Figure 1). In the upstream pathway, phenylalanine is transformed into glycyrrhizin and naringin through the continuous action of phenylalanine ammonia-lyase (PAL), trans-cinnamate 4-monooxygenase (C4H), 4-coumaric acid CoA ligase (4CL), chalcone synthase (CHS) and chalcone isomerase (CHI) [4,11]. In the downstream pathway, Isoflavonoids are generated from liquiritigenin or naringenin under the hydroxylation, methylation and glycosylation by 2-hydroxyisoflavanone synthase (IFS), 2-hydroxyisoflavanone dehydratase (HIDH), methyltransferases, and glycosyltransferases, respectively [4,7].

Before the 2005 edition of the *Chinese Pharmacopoeia*, *P. lobata* and *P. thomsonii* were regarded as Pueraria and as a source of traditional Chinese medicine. In the long-term process of introduction, cultivation and domestication, the names and plant species of *P. lobata* and its varieties are confused in most areas and studies, resulting in the unclear resource location of *P. lobata* and its varieties [3]. In terms of composition, the root of *P. lobata* usually contains a high content of puerarin, up to 60–70 mg/g, while the puerarin content of *P. thomsonii* is usually less than 5 mg/g [12]. In terms of genetic material, the genomes of the two kinds of *P. lobata* have also changed greatly. At present, many research reports are available on the transcriptome and related isoflavone synthesis genes of *P. lobata* or *P. thomsonii*. Additionally, there are two reports on the genome of *P. thomsonii* and *P. montana* [13,14], but the data on the genome of *P. lobata* have not been reported [10]. The lack of reference genome information on *P. lobata* hinders further investigation of potential genes in important biological processes related to isoflavone biosynthesis [10,15].

In this study, the leaves of *P. lobata* were chosen as the material, and the genome sequence of *P. lobata* was obtained and assembled by PacBio and Illumina Hiseq sequencing technology. After completing assembly and annotation, we explored the unique genes of isoflavones and puerarin metabolism in legumes through genome-wide comparison, gene family clustering, phylogenetic tree and other methods, and explored the relationship between puerarin-specific genes and special biological traits. The evolutionary status of *P. lobata* species was identified, and the genome evolutionary history of species and even the whole branch was traced. The study further revealed the pathway and mechanism of puerarin synthesis in *P. lobata* by a transcriptome and metabolome analysis. This research expected to provide reference data support for the comprehensive utilization and classification of medicinal plant resources of *P. lobata* and *P. thomsonii*.

## 2. Materials and Methods

### 2.1. Materials, DNA Extraction and Genome Sequencing

The diploid *P. lobata* were planted in an experimental medicinal botanical garden at Wuhan Polytechnic University (Longitude: 114.23855 latitude: 30.63535, Wuhan, China) and Luotian (31.021052, 115.595175). The roots of four cultivated varieties of *P. lobata* with varying puerarin levels, PlobLT13, Plob53, Plob17, Plob19 and Plob25, were utilized for the transcriptome and metabolome analysis, and were marked as A, B, C, D and E, respectively, in this research (Table S1). Root, stem, young leaves, flower and seeds of *P. lobata* were collected and immediately frozen in liquid nitrogen and stored at −80 °C until use for DNA and RNA extraction.

Genomic DNA was extracted from young leaves of *P. lobata* and used to construct Illumina DNA libraries according to the standard protocols provided by the Illumina HiSeq company (Novogene Biotech Co., Ltd., Tianjin, China). A PacBio library was constructed using a SMRTbell Template Prep Kit 1.0 (PacBio, Menlo Park, CA, USA) and sequenced on a PacBio Sequel II system.

### 2.2. Genome Assembly and Quality Evaluation

All DNA extraction and sequencing procedures were performed by the Novogene Company (Tianjin, China) (http://www.novogene.com/, accessed on 31 October 2022). The Hi-C library was sequenced on the Illumina NovaSeq PE150 platform. PacBio readings were utilized for de novo assembly, and Pilon v1.22 was used to polish them using Illumina data [16]. The corrected contigs were further scaffolded into chromosomal-level genome via Hi-C. Hi-C technology obtained the interaction information between DNA fragments that were spatially connected, that is, DNA fragments that were physically distant, through special experimental techniques. Different contigs or scaffolds were divided into different chromosomes according to the probability of interaction within chromosomes being significantly higher than the probability of interaction between chromosomes. According to the same chromosome, the probability of interaction decreased with the increase in the interaction distance. Contigs or scaffolds of the same chromosome were sorted and oriented. Hifiasm software (v0.16.1) was used for the rapid construction of haplotype from the scratch assembly program of PacBio Hifi reads [17,18]. We used Samtools (v0.1.19) (http://samtools.sourceforge.net/, accessed on 2 October 2022) and other tools to sort. The BWA (v0.7.8) comparison resulted in chromosome coordinates, removing duplicate reads, etc., conducting SNP calling, and filtering and counting the original results. BUSCO (v5.2.1) was applied to evaluate the completeness of assembly by mapping the genome sequence to the embryophyta_odb10 database [19]. CGEMA (v2.5) was used to evaluate the integrity of the assembled genome. The conservative genes (248 genes) existing in 6 eukaryotic model organisms were selected to form the core gene library, and the assembled genome evaluated with tblastn, genewise and geneid software [20]. Based on the K-mer algorithm, Illumina sequencing data and Merqury software (https://github.com/marbl/merqury, accessed on 25 June 2022) were used to evaluate genome quality, which did not need to reference genome [21]. The genome assembly software utilized in this work is listed in Table S2.

### 2.3. RNA-seq Data

Trinity (v2.1.1) was used to construct transcriptome read assemblies for genome annotation. To optimize the genome annotation, RNA-Seq reads from various tissues were aligned to the fasta genome using Hisat (v2.0.4) and TopHat (v2.0.11) with default parameters to identify exons regions, and splice sites. The alignment results were then utilized as input for genome-based transcript assembly using Stringtie (v1.3.3)/Cufflinks (v2.2.1) with default settings. The non-redundant reference gene collection was created by combining genes predicted by three ways with EvidenceModeler (EVM, v1.1.1), adding masked transposable elements as input into gene prediction and employing PASA (Program to Assemble Spliced Alignment) terminal exon support. Individual families of interest were hand-picked by relevant specialists for further human curation.

### 2.4. Genome Annotation

In our repeat annotation workflow, we used a combination technique based on homology alignment and de novo search to find entire genome repetitions. Tandem Repeat was retrieved using ab initio prediction and TRF (http://tandem.bu.edu/trf/trf.html, accessed on 25 June 2022). The Repbase (http://www.girinst.org/repbase, assessed on 2 October 2022) database was used for homolog prediction, and it employed RepeatMasker (http://www.repeatmasker.org/, assessed on 2 October 2022) software and its in-house scripts (RepeatProteinMask) with default settings to extract repeat regions. Then, using LTR FINDER (http://tlife.fudan.edu.cn/ltrfinder/, assessed on 2 October 2022), RepeatScout (http://www.repeatmasker.org/, accessed on 2 October 2022) and RepeatModeler (http://www.repeatmasker.org/RepeatModeler.html, assessed on 2 October 2022) with default parameters, all repeat sequences with lengths >100 bp and gap ‘N’ less than 5% comprised the raw transposable element (TE) library. For DNA-level repeat detection, a bespoke library (a mix of Repbase and our Denovo TE library processed by uclust to create a non-redundant library) was submitted to RepeatMasker.

To annotate gene models, structural annotation of the genome was employed, which includes AB initio prediction, homology-based prediction and RNA-Seq aided prediction.

The homologous protein sequences were obtained from NCBI. Protein sequences were matched to the genome using TBLASTN (v2.2.26; E-value 10^5^), and the matching proteins were aligned to the homologous genome sequences for accurate spliced alignments using GeneWise (v2.4.1) software, which predicted the gene structure present in each protein region.

Augustus (v3.2.3), Geneid (v1.4), Genescan (v1.0), GlimmerHMM (v3.04) and SNAP (29 November 2013) were applied in our automated gene prediction pipeline for Ab initio gene prediction.

By matching the protein sequences to the Swiss-Prot database using BLASTP (with a threshold of E-value ≤ 10^−5^), gene functions were given based on the best match. InterPro Scan 70 (v5.31) was used to annotate the motifs and domains by searching against publicly available databases such as PRINTS, Pfam, ProDom, PANTHER, SMRT and PROSITE. Each gene’s Gene Ontology (GO) ID was allocated based on the corresponding InterPro entry. We predicted protein function by transferring annotations from the nearest BLAST hit (E-value 10^−5^) in the Swiss-Prot 20 database and DI-AMOND (v0.8.22)/BLAST hit (E-value 10^−5^) in the NR 20 database. We also mapped the gene collection to a Kyoto Encyclopedia of Genes and Genomes (KEGG) pathway and determined which genes were the best matches.

The tRNAs were predicted using the tRNAscan-SE tool (http://lowelab.ucsc.edu/tRNAscan-SE/, assessed on 2 October 2022). Because rRNAs are highly conserved, we used relative species’ rRNA sequences as references and used BLAST to predict rRNA sequences. Other ncRNAs, including as miRNAs and snRNAs, were discovered by searching the Rfam database with the default parameters of the infernal program (http://infernal.janelia.org/, assessed on 2 October 2022).

### 2.5. Gene Family and Phylogenomic Analysis

To identify gene family groups, protein-coding genes from 14 species, *Pueraria_lobata*, *Arabidopsis_thaliana*, *Glycine_max*, *Medicago_truncatula*, *Arachis_duranensis*, *Arachis_hypogaea*, *Phaseolus_vulgaris*, *Cajanus_cajan*, *Cicer_arietinum*, *Vigna_radiata*, *Trifolium_pratense*, *Lupinus_albus*, *Vigna_unguiculata* and *Vigna_angularis* were analyzed (Table S3).

The longest transcript in the coding region was retained to remove redundancy, and the genes encoding polypeptides shorter than 30 amino acids were also abandoned to exclude putative fragmented genes. The similarity relation between all species’ protein sequencings was obtained by All-against-all BLASTP (https://blast.ncbi.nlm.nih.gov/Blast.cgi/, assessed on 2 October 2022) [22] search with a cut-off (E-value = 10^−5^). The alignment with high-scoring segment pairs was conjoined for each gene pair by solar [23]. A hierarchical clustering algorithm was applied to group orthologs and paralogs using OrthoMCL software (http://orthomcl.org/orthomcl/, assessed on 2 October 2022) [24] with the inflation parameter 1.5.

### 2.6. Phylogenetic Analysis

Single-copy orthologous genes were aligned with Muscle (http://www.drive5.com/muscle/, assessed on 2 October 2022) [25]. A super alignment matrix was created by concatenating all alignment findings. RAxML was used to generate ML phylogenetic trees based on multiple sequence alignments [26].

### 2.7. Divergence Times Estimate

Divergence times were calculated using single-copy orthologous by the MCMC Tree program of PAML (http://abacus.gene.ucl.ac.uk/software/paml.html, assessed on 2 October 2022) [27] with main parameters (burn-in = 10,000, sample-number = 100,000, sample-frequency = 2). The calibration times were taken from the TimeTree database (http://www.timetree.org/, assessed on 2 October 2022) [28].

### 2.8. Gene Family Expansion and Contraction

To identify gene family evolution as a random birth and death process model, where gene family either expands or contracts per gene per million years independently along each lineage of the phylogenetic tree, the maximum likelihood model originally implemented in the software package CAFE (http://sourceforge.net/projects/cafehahnlab/, assessed on 2 October 2022) was applied to compare the cluster size differences (gain or loss) between the ancestor and each species [29]. A *p*-value of 0.05 was used to identify families where the size of the species has changed considerably. To determine the relevance of changes in gene family size in each branch, the phylogenetic tree topology and branch lengths were considered.

### 2.9. Positively Selected Genes

Single-copy orthologous genes were aligned with Muscle (http://www.drive5.com/muscle/, assessed on 2 October 2022) [25]. Likelihood ratio tests (LRTs) based on the branch-site model of PAML were used to detect positive selection sites with *A. thaliana* as the foreground branch [30]. The *p*-values were computed using the χ^2^ statistic and corrected for multiple testing by the false discovery rate (FDR) method. This analysis calculated Ka/Ks through a likelihood ratio test to detect the probability of positive selection.

### 2.10. Whole-Genome Duplication Inference

An all-against-all BLASTP (https://blast.ncbi.nlm.nih.gov/Blast.cgi/, assessed on 2 October 2022) was used with a threshold (E-value = 10^−5^) to identify the putative paralogous genes in, and orthologous genes between, each species. Syntenic blocks were performed based on the detected homologous gene pairs using MCscanX (http://chibba.pgml.uga.edu/mcscan2/, assessed on 2 October 2022) [22,30]. Each duplicate gene pairs of syntenic block were aligned with Muscle [25], and then back-translated to their coding sequences. The four-fold synonymous third-codon transversion rates (4DTv) of syntenic blocks were calculated and were used to detect WGD events. The distribution of 4DTv values was plotted. The synonymous substitution (Ks) values for pairwise comparisons were estimated using the maximum likelihood (ML) method implemented in the codeml program of the PAML package [27].

### 2.11. WGCNA Analysis

A Weighted Gene Co-expression Network Analysis (WGCNA) was performed by the Novogene online tools to discover critical regulatory genes in the correlation between puerarin production and gene expression in various species (https://magic.novogene.com/customer/, assessed on 2 October 2022). The module eigengene was defined as the first major component of a specific module and was then used to describe the expression profile of module genes in each sample. The Pearson correlations between the eigengenes of each module and the abundance of flavonoids were performed using R package ggplot2.

### 2.12. Metabolome Analysis

The roots of different *P. lobata* samples were used for the metabonomic analysis (Table S1). The metabolome analysis was completed by Metware Biotech Co., Ltd. (www.metware.cn. Wuhan, China) according to the methods of Cheng [31]. The freeze-dried roots were crushed using a mixer mill (mm 400, retsch) with a zirconia bead for 1.5 min at 30 Hz. Then, 100 mg powder was weighted and extracted overnight at 4 °C with 1.0 mL 70% aqueous methanol. Following centrifugation at 10,000 g for 10 min, the extracts were absorbed (CNWBOND Carbon-GCB SPE Cartridge, 250 mg, 3 mL; ANPEL, Shanghai, China) and filtrated (SCAA-104, 0.22 μm pore size; ANPEL, Shanghai, China) before the LC-MS analysis.

The sample extracts were analyzed using an LC-ESI-MS/MS system (HPLC, Shim-pack UFLC SHIMADZU CBM30A system, www.s himadzu.com.cn/ (assessed on 2 October 2022); MS, Applied Biosystems 6500 Q TRAP, www.a ppliedbiosys-tems.com.cn/, assessed on 2 October 2022). The analytical conditions were as follows, HPLC: column, Waters ACQUITY UPLC HSS T3 C18 (1.8 µm, 2.1 mm × 100 mm); solvent system, water (0.04% acetic acid): acetonitrile (0.04% acetic acid); gradient program, 95:5 *v/v* at 0 min, 5:95 *v/v* at 11.0 min, 5:95 *v/v* at 12.0 min, 95:5 *v/v* at 12.1 min, 95:5 *v/v* at 15.0 min; flow rate, 0.40 mL/min; temperature, 40 °C; injection volume: 2 μL. The effluent was alternatively connected to an ESI-triple quadrupole-linear ion trap (Q TRAP)-MS.

The ESI-Q TRAP-MS/MS. LIT and triple quadrupole (QQQ) scans were acquired on a triple quadrupole-linear ion trap mass spectrometer (Q TRAP), API 6500 Q TRAP LC/MS/MS System, equipped with an ESI Turbo Ion-Spray interface, operating in a positive ion mode, and controlled by Analyst 1.6.3 software (AB Sciex).

### 2.13. Statistical Analysis

Excel 2021 and SPSS (22.0) were used to process experimental data. Chen’s approach was used to process the heat map, Circos, chromosomal collinearity and related network diagram content analysis using the TB-tools software. Duncan’s test was performed to see whether there were any significant changes (*p* ≤ 0.05) [32]. The correlation network was created with the OmicStudio tools, which may be found at https://www.omicstudio.cn/tool (assessed on 2 October 2022). The positive correlation criterion was greater than or equal to 0.5, the negative correlation threshold was less than or equal to −0.5 and the *p*-value threshold was less than 0.5. R version 3.6.1 and igraph1.2.6 [31]. Every experiment had three biological duplicates.

## 3. Results

### 3.1. Genome Assembly and Annotation

**Figure 2:**
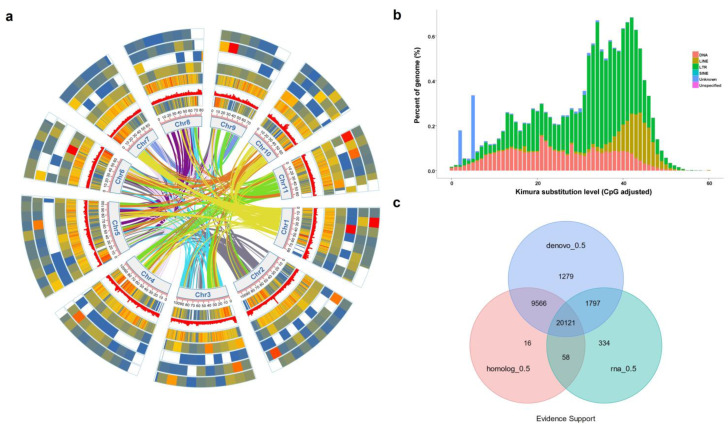
Basic structural features of *P. lobata* genome, annotated genes. (**a**) Genomic landscape of the 11 assembled pseudochromosomes. Track i represents the length of the pseudochromosomes (Mb); ii–iv represent distribution of gene density, all repeat element and tandem repeat, respectively; and v–viii show the distribution of miRNA, tRNA, rRNA and snRNA, respectively. (**b**) The abscissa is the divergence degree between transposition element (TE) sequences annotated in *P. lobata* genome and corresponding sequences in Repbase. The ordinate is the percentage of TE sequence in the genome under the divergence degree, and different TE is marked with different colors. (**c**) Gene set evidence supporting statistics, de novo, EVM integrate genes supported by de novo prediction; homolog, genes supported by homology prediction during EVM integration; RNA, RNA SEQ supported genes during EVM integration; each evidence support is based on the criterion that the gene overlap is greater than 50%; numbers indicate the number of genes. Following the assembly assisted by Hi-C technology, 97.82% of the contigs were anchored into 11 pseudochromosomes, resulting in a chromosome-scale genome with a size of 942.73 Mb in *P. lobata* (Figure 2, Table S5). The lengths of contig N50, scaffold N50 and the longest scaffold are 29.51 Mb, 29.51 Mb and 60.01 Mb, respectively, indicating high continuity of the assembled gnome (Table S6).

The *P. lobata* genome was sequenced using the Illumine Hiseq and PacBio sequel platforms, and the assembled scaffolds were ordered using the Hi-C technique. The project obtained 84.61 Gb data, of which 57.99 Gb data were obtained from Illumina second-generation sequencing and 26.62 Gb data from Pacbio third-generation sequencing. The genome sequencing coverage reached 91.18% (Figure 2a and Table S4). The genome size calculated by the KMER number/depth is about 942.73 Mbp, and the revised genome size is 939.2 Mbp, with GC contents of 33.69%, further clustered into 11 pseudochromosomes (Figure S1, Table 1). The assembled version was evaluated by various methods, and the results showed that the genome consistency, integrity and accuracy were good.

The BUSCO evaluation results showed that the genome of *P. lobata* had assembled 99.2% of the complete BUSCO gene, indicating that the assembly result was complete. In addition, the CEGMA evaluation showed that 248 CEGs (Core Eukaryotic Genes) assembled 240 genes, accounting for 96.77%, indicating that the assembly results were complete (Table S6). Sequence consistency evaluation results showed that the alignment rate of small reads to genome was about 98.79%, and the genome coverage was about 99.97%, indicating that reads and the assembled genome had good consistency (Table S7). The heterozygous SNP ratio of the *P. lobata* genome was 0.468784%, and the homozygous SNP ratio was 6.9 × 10^−5^%. This result shows that the assembly had a high single base correct rate (Table S8). The results of sequence accuracy evaluation showed that the accuracy value of the genome sequence was 46.0206, indicating that the accuracy of this genome was good.

The repeat sequence statistical results showed that the proportion of repeat was 63.5% in the genome of *P. lobata*. According to the statistics of repeat sequences, the proportions of Denovo + RepBase, TE proteins and Combined TEs were 61.98%, 7.91% and 62.42%, respectively (Tables S9 and S10). Taking RepBase as the library, the TE divergence distribution was obtained by the Repeat Masker annotation. The abscissa was the divergence between the TE sequence annotated in *P. lobata* genome and the corresponding sequence in the RepBase. The ordinate was the percentage of TE sequences in the genome under this divergence, and different TEs were marked with different colors (Figure 2b).

**Figure 3:**
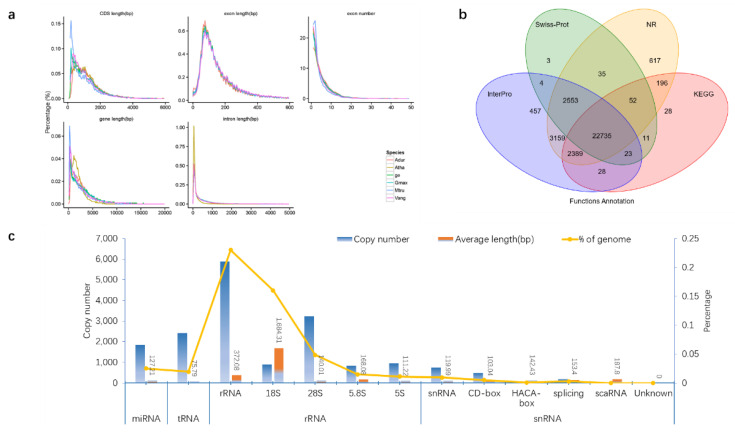
Gene structure and function annotation of *P. lobata* genome. (**a**) Statistical information of gene structure and comparative information of various components of *P. lobata*(ge), *Vigna angularis* (Vang), *Glycine max* (Gmax), *Medicago truncatula* (Mtru), *Arabidopsis thaliana* (Atha), *Arachis duranensis* (Adur). (**b**) The protein sequences predicted by gene structure were aligned with known protein libraries (Swiss_Prot, Nr, KEGG, InterPro, GO and Pfam), and finally 97.34% of structural genes were able to predict the function. (**c**) By comparing with the known ncRNA library, the ncRNA distribution information of *P. lobata* genome was obtained. miRNA can degrade its target genes or inhibit the translation of target genes into proteins and has the function of silencing genes. tRNA and rRNA are directly involved in protein synthesis. snRNA is mainly involved in the processing of RNA precursors and is the main component of RNA shears.

*Vigna angularis*, *G. max*, *Medicago truncatula*, *Arabidopsis thaliana* and *Arachis duranensis* were selected as annotation species, and the genome of *P. lobata* was annotated with the software Augustus, Glimmerhmm, SNAP, GeneID and GENSCAN. The results showed the *P. lobata* genome sequence had 27,356, 27,237, 30,958, 24,987 and 35,325 structural genes homologous with *V. angularis*, *A. duranensis*, *M. truncatula*, *A. thaliana* and *G. max*, respectively (Figure 2c and Table S11). Compared with the annotated species, the average transcript length of genomic structural genes of *P. lobata* was 3785.5 bp, the average CDS length was 1170.28 bp, the average exons of each gene were 4.98 and the average length was 234.81 bp. The average intron length was 656.43 bp (Figure 3a and Table S12).

Compared with the existing protein library, 97.34% of the structural genes in the *P. lobata* genome can predict their functions. Compared with the existing protein database, 32,190 of 33,171 structural genes in *P. lobata* genome can predict the function, accounting for 97.34%; 881 structural genes were not annotated, accounting for 2.66% (Figure 3b and Table S13).

The statistical analysis of non-coding RNA in the *P. lobata* genome showed that miRNA, tRNA, rRNA and snRNA accounted for 0.024961%, 0.019503%, 0.23% and 0.009531% of the genome sequence, respectively (Figure 3c and Table S14).

### 3.2. Phylogenetic Relationships and Comparative Genomic Analysis

**Figure 4:**
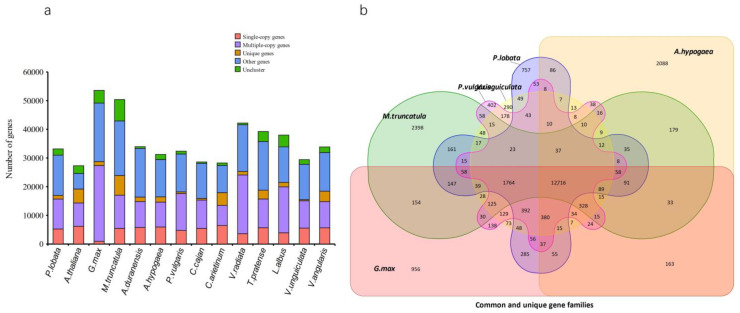
Comparative analysis of genes and gene families among different species. (**a**) The distribution of different species. This is a stacked column chart of the clustering statistical results of gene families of each species, in which the horizontal axis is the name of the species, and the vertical axis is the number of corresponding genes. According to the clustering results, the genes of each species are divided into the following five categories. Single-copy genes (red): the number of species contained in the gene family is equal to the total number of species, and the number of genes of a species is 1; multiple-copy genes (purple): the number of species contained in the gene family is equal to the total number of species, and the number of genes of a species is greater than 1; unique genes (yellow): the number of species contained in the gene family is equal to 1, and the family is composed of a single species; other genes (blue): families in which the number of species contained in the gene family is greater than 1 and less than the total number of species; unclustered genes (green): genes that do not belong to any of the above four categories are unclustered; (**b**) common and unique gene families. The overlapped part between circles in the figure represents the number of gene families common among species, the non-overlapped part represents the number of gene families unique to this species and the sum of the numbers in a complete circle represents the total number of gene families of this species.

Through the cluster analysis of gene families, the results showed a total of 30,367 orthologous groups with representatives in the 14 species, of which 9449 were shared orthologs and 160 were single-copy orthologs (Figure 4a). The corresponding clustering results of the genomes of *P. lobata*, *P. vulgaris*, *V. unguiculata*, *G. max*, *M. truncatula* and *A. hypogaea* were extracted, and the Venn diagram was drawn. The Venn diagram showed that compared with other species, *P. lobata* comprised 757 species-specific gene families, including 1874 genes. These included 285 unique gene families between *P. lobata* and *G. max* (Figure 4b).

**Figure 5:**
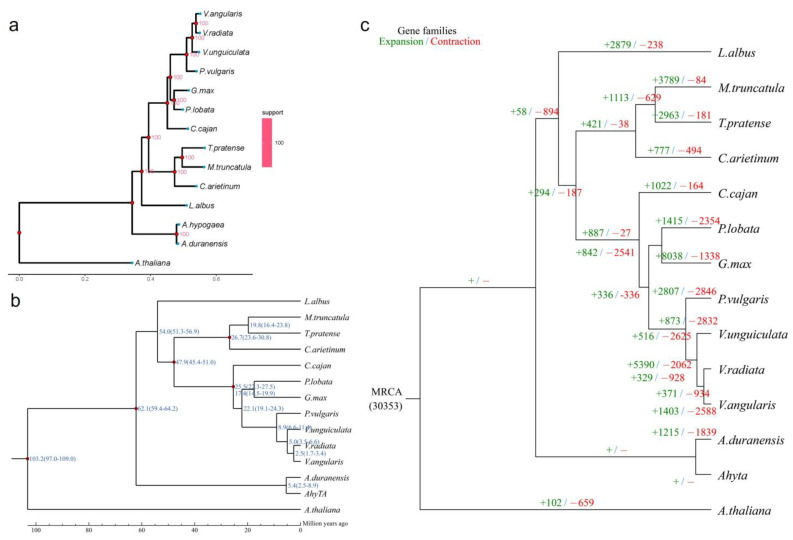
Analysis of species evolution and estimation of species divergence time among legumes. (**a**) Phylogenetic tree. The evolutionary tree is composed of branches, branch lengths and nodes. Branches: each branch corresponds to a species (blue end point); branch length: the length of each black horizontal line, which represents the speed of species evolution. The longer it is, the faster it evolves. The lowest black ruler can measure the branch length; node: the intersection of two branches represents the ancestor node of two branches, which is represented by red solid points in the figure. The number next to each solid point represents the support rate. The larger the value is, the more reliable the evolutionary relationship is. The heat map on the far right is the heat map of the support rate of all branches in the evolutionary tree; (**b**) estimation of divergence time. The number of the node position represents the divergence time of species or species ancestors, in millions of years. The number in brackets represents the confidence range of the divergence time; (**c**) expansion and contraction in gene families. Green numbers indicate the number of gene families that have expanded during the evolution of species, and red numbers indicate the number of gene families that have contracted.

Phylogenetic trees of *P. lobata* and other leguminous plants were constructed by the maximum likelihood method (ML TREE). Among the 14 species selected for comparison, *P. lobata* and *G. max* had the most recent evolutionary relationship (Figure 5a).

The single-copy gene family estimated the time of species differentiation through PAML and MCtree software packages. The results showed that the divergence time correction points of *P. lobata* and *G. max* were 17.4 million years (Mya), while those of the other species were 25–45 Mya between *C. arietinum* and *T. pratense*, 20.9–27.1 Mya between *V. angularis* and *V. radiata*, 46–60 Mya between *V. unguiculata* and *V. radiata* and 59–64 Mya between *A. hypogaea* and *A. duranensis*. The time correction points were taken from the Timetree (http://www.timetree.org/, assessed on 2 October 2022) (Figure 5b).

The expansion and contraction analysis showed that the most recent common ancestor had 30,353 gene families. Compared with their recent ancestors, *P. lobata* significantly expanded 1415 gene families (including 6641 genes) and significantly contracted 2354 gene families, including 472 genes (Figure 5c). In the isoflavone metabolic gene family of *P. lobata*, 41 genes were expanded, and 35 genes were enriched in the molecular function pathway; 7 genes contracted and were all enriched in the molecular function pathway (Figure S2).

### 3.3. Positive Selection and WGD Analysis

Taking *P. lobata* as the foreground branch of the positive selection analysis, and other species *M.truncatula*, *G. max*, *P. vulgaris*, *V. unguiculata* and *A. hypogaea* as the background branch, 95 candidate genes subject to positive selection were found through the likelihood ratio test (Q-value < 0.05). The Fisher test and FDR correction (Q-value < 0.05) were used to analyze the functional enrichment of these positively selected genes. These were enriched in the GO terms of RNA processing, hydrolase and nuclease activity (Figure S3a), and in KEGG terms, including cell cycle, Fanconi anemia and ABC transporter (Figure S3b). In the positive selection event, the flavonol 7-O-beta-glucosyltransferase (Plob17707_Plob) gene related to puerarin synthesis was preserved and expanded in evolution.

**Figure 6:**
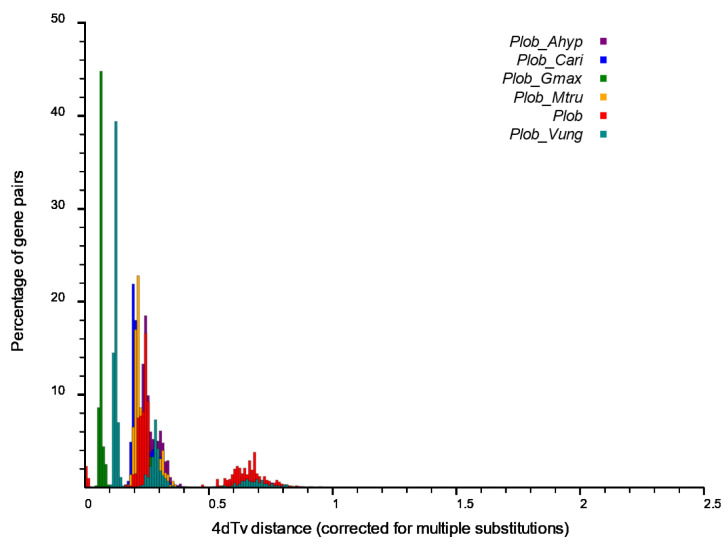
Comparative analysis of 4dTv distance between *P. lobata* and five other legumes. The horizontal axis is the value of 4dTv, and the vertical axis represents the proportion of corresponding gene pairs. The size of 4dTv value on the horizontal axis reflects the sequence of time (the greater the 4dTv value, the longer the time is. The more obvious the 4dTv value, the closer the time is to the present).

Five legumes, including *M.truncatula*, *G. max*, *C. arietinum*, *V. unguiculata* and *A. hypogaea* were compared with *P. lobata* for the Muscle multiple sequence alignment to further analyze the WGD events that occurred in the evolution of *P. lobata*. Based on the alignment results, the 4DTv value was calculated, and the frequency distribution map of 4DTv and gene pairs was drawn. The smaller the abscissa value, the closer it was to the present, and the larger the value, the older it was. The peak within species indicated the WGD, and the peak between species indicated species differentiation. This distribution map showed that *P. lobata* had two WGDs, about 0.65 once (a gamma event shared by the core dicotyledons), and about 0.25 once (WGD shared by Leguminosae before differentiation with several Leguminosae species), and about 0.05 separated from the nearest *G. max*. Since then, its own WGD has not occurred (Figure 6).

### 3.4. Collinearity Analysis of P. lobata and Other Leguminous Plants

**Figure 7:**
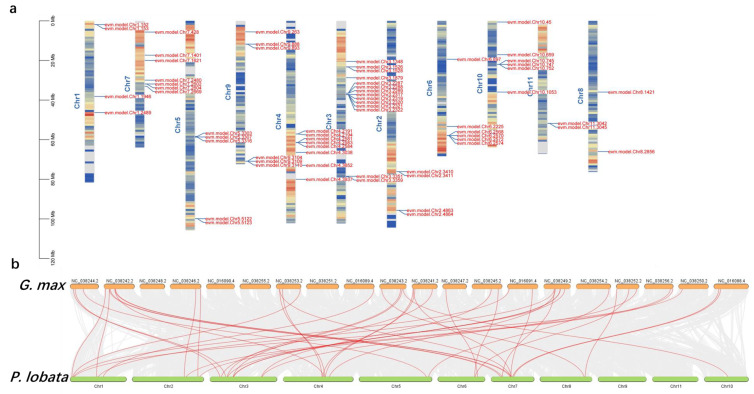
Collinearity analysis of *P. lobata* and *G. max* genome. (**a**) Distribution of genes related to puerarin metabolic pathway on the chromosomes of *P. lobata*. The red color represents the larger gene density, and the blue color represents the smaller gene density. (**b**) Massive collinear blocks were identified among *P. lobata* and *G. max*, which indicated well-preserved genome structures in this family. The red lines are marker genes related to puerarin anabolism.

We selected five legume genomes for a comparative analysis to examine the chromosome evolution of *P. lobata* and other legumes. Massive collinear blocks were identified among Leguminous plants, which indicated well-preserved genome structures in this family. A total of 36.47% (48,807 gene pairs), 61.45% (54,579), 42.38% (35,409 gene pairs), 63.96% (39,014) and 63.13% (39,516) of the *P. lobata* genome was syntenic with *A. hypogaea*, *G. max*, *M.truncatula*, *P. vulgaris* and *V. unguiculata*, respectively (Figure 7, Figure S4). Compared with the *G. max* genome, six unique *PlUGT43* homologous genes were retrieved from the *P. lobata* genome (Figure S5).

### 3.5. Combined Transcriptome and Metabolome Analysis Reveals the Metabolic Mechanism of Puerarin

**Figure 8:**
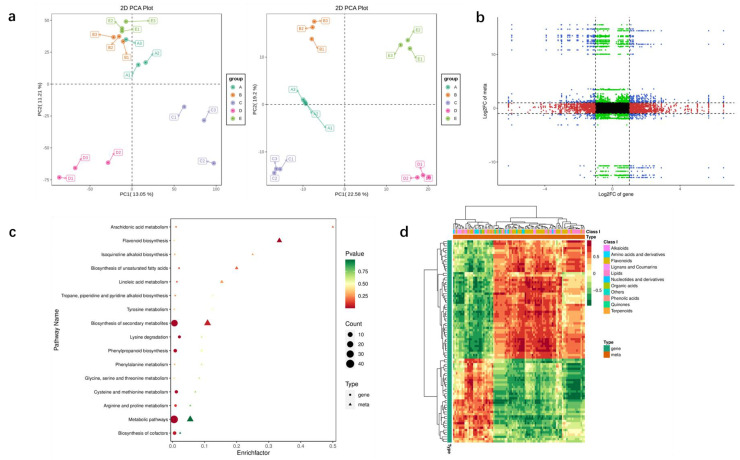
Combined analysis of transcriptome and metabolome of *P. lobata* samples with different puerarin contents. (**a**) PCA of metabolites and transcriptome. The abscissa table represents principal component 1, the ordinate represents principal component 2 and the dots of different colors represent samples of different groups. (**b**) Nine quadrant diagram of differential metabolites and genes. The difference multiples of substances with Pearson’s correlation coefficient greater than 0.80 and *p*-value less than 0.05 in each difference group are shown by the 9 quadrant diagram, which is divided into 1–9 quadrants from left to right and from top to bottom with black dotted lines. The abscissa represents log2FC of genes and the ordinate represents log2FC of metabolites. (**c**) KEGG enrichment analysis of differential metabolites and expressed genes. The abscissa represents the enrichment factor of this pathway in different omics, and the ordinate represents the name of KEGG pathway; The steady transition from red to yellow to green shows the shift in the significance of enrichment from high to medium to low., expressed by *p*-value; the shape of bubbles represents different omics, and the size of bubbles represents the number of different metabolites or genes. The larger the number, the larger the dot. (**d**) Correlation heat map of differential genes and metabolites. Each row is a gene, and each column is a metabolite. Red represents a positive correlation between genes and metabolites, and green represents a negative correlation between genes and metabolites.

In this experiment, five *P. lobata* cultivars were selected, and the content of total flavonoids in their roots was significantly different. The content of total flavonoids in samples A, B, C, D and E was 7.13%, 7.96%, 11.57%, 9.34% and 10.21%, respectively (Table S1). The transcriptome and metabolome analysis of *P. lobata* was tested from different cultivars to better explore the relationship between the puerarin synthesis and related metabolic genes (Figure 8). The PCA analysis results of metabolome and transcriptome data showed that the correlation of biological duplicate samples within genotypes was much greater than that between genotypes, which proved the reliability of experimental sample data. We selected the group with the largest differential metabolites and differentially expressed genes (DEGs) for the comparative analysis. The heat map of direct correlation between DEGs and differential metabolites showed that isoflavones and flavonoid metabolites had highly correlated with expressed genes. The results showed that there were 934 different metabolites in group A and group C, including 223 flavonoids and 42 isoflavones. Among the differential metabolites, isoliquiritigenin, liquiritigenin, daidzin, daidzein, puerarin and puerarin-4′-o-glucoside which are unique to *P. lobata*, were detected (Table S15). A total of 348 DEGs were identified in the 2 groups, including GLYMA_01G232400 4-coumarate CoA ligase (*4CL*), GLYMA_ 18G285800 chalcone reductase (*CHR*), GLYMA_03G181600 and GLYMA_ 19G182300 (*PAL*), which were positively correlated with the synthesis of liquiritigenin, daidzein and puerarin, and the correlation coefficient was higher than 0.8 (Table S16).

### 3.6. WGCNA Analysis of DEGs

**Figure 9:**
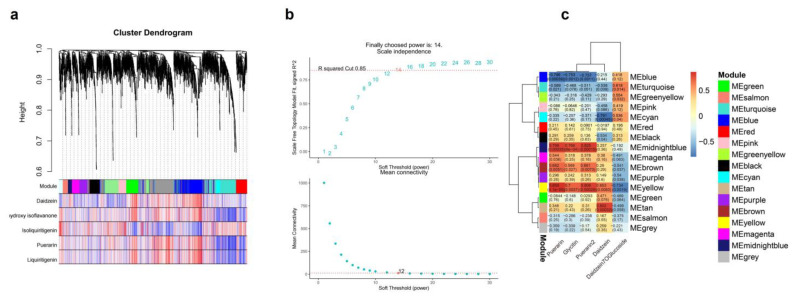
WGCNA analysis between puerarin metabolism difference and transcriptome data. (**a**) The WGCNA constructed by dynamic tree cutting method, different modules are marked with different colors. (**b**) The correlation between modules and traits. (**c**) The scale-free network fitting index (*R*^2^) under different soft thresholds; the red line represents *R*^2^ = 0.85. The average connectivity under different soft thresholds.

The WGCNA was performed on the expressed genes of four *P. lobata* samples with large differences in the isoflavone content to further identify the gene co-expression modules of isoflavone and puerarin metabolism in *P. lobata*. After filtering out the low-expression genes, 19,163 genes were reserved for network construction. Figure 9a,b show the fitting index and average connectivity under different soft thresholds. The analysis set the fitting index as *R^2^* = 0.85, and the weighting coefficient β = 14 and the scale-free network construction was carried out.

The clustering of co-expressed genes was dynamically cut, and 16 modules were generated after combining similar clusters. The genes contained in the gray module showed chaotic expression patterns, which were not considered in the subsequent co-expression analysis. Therefore, we finally determined 15 co-expression modules. According to the gene module cluster tree (Figure 9a), all co-expression modules were divided into two categories, and two different modules had different degrees of correlation. In the correlation heat map (Figure 9c) of the module, blue represented negative correlation, and the closer the value was to 0, the greater the degree of negative correlation. Red represented positive correlation, and the closer the value was to 1, the greater the positive correlation.

The WGCNA analysis yielded 15 modules with similar gene expression patterns, each containing 5 to 432 genes (Figure 9a). Figure 9b shows the correlation analysis between the module characteristic genes and five isoflavones (daidzein, 2,7,4′-trihydroxyisoflavone, isoliquiritigenin, puerarin and liquiritigenin). As shown in the figure, the midnight blue and yellow modules were positively correlated with the content of puerarin, while the blue module negatively correlated with the content of puerarin. The characteristic genes of these three modules highly correlated with the accumulation of puerarin. Figure 9c shows the correlation network diagram between the module characteristic genes and isoflavones and puerarin metabolites. The diagram shows that the turquoise module had a high correlation at various concentrations in various parts.

**Figure 10:**
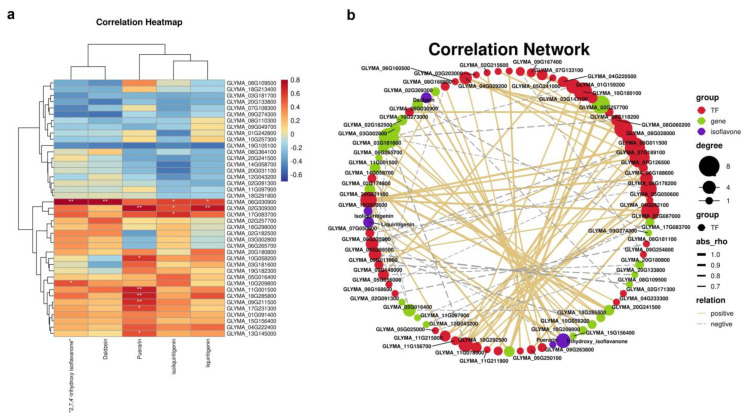
The correlation heat map and network relationship between gene expression patterns and four different isoflavone forms contents in Blue and Grey60 modules. (**a**) Heat map of correlation coefficient between different isoflavone forms’ contents and module characteristic genes; red and blue blocks represent positive and negative correlation, respectively. (**b**) Regulating network between the selected genes and four different isoflavone forms’ compounds in *P. lobata*. The positive correlation threshold is set to be greater than or equal to 0.5, the negative correlation threshold is set to be less than or equal to −0.5 and the *p*-value threshold is less than 0.5, R version 3.6.1, igraph1.2.6.

The core genes and transcription factors related to isoflavone metabolism were screened out from the modules, and the Pearson correlation analysis was carried out between these genes and transcription factors and five isoflavones (daidzein, 2,7,4′-trihydroxyisoflavone, isoliquiritigenin, puerarin and liquiritigenin). Figure 10a shows the correlation heat map between the module characteristic genes and isoflavones and puerarin metabolites. As shown in the figure, the turquoise module had a high correlation in all parts and concentrations. Figure 10b shows the network relationship between isoflavone synthesis-related genes, transcription factors (TFs) and puerarin isoflavones. From the midnight blue and yellow modules, we screened 68 TFs positively related to the synthesis of puerarin and isoflavones. The results showed that *MYBs* (GLYMA_06g160500 and GLYMA _11g215800), *WRKYs* (GLYMA_08g118200 and GLYMA_09g254800), *bHLH* (GLYMA_02g174800) and other TFs were involved in the synthesis of puerarin.

## 4. Discussion

*P. lobata* is an important medicinal and edible homologous plant that is widely cultivated in Asian countries [33]. The stem skin fiber of *P. lobata* is often used as a raw material for weaving and papermaking in the industry [3]. *P. lobata* has been considered as an important traditional Chinese medicine and homologous food for hundreds of years, with economic market potential. Its roots are not only nutritious, but also have many pharmacological properties, including flavonoids and isoflavones, which are widely used to treat and prevent various diseases [10,34]. In the query of specimens, it is found that the identification of *P. lobata*, *P. thomsonii* and *P. montana* in most specimens is confusing, especially in the older specimens, where many of them identify *P. thomsonii*, and *P. montana* as *P. lobata*. At present, only *P. lobata* and *P. thomsonii* are used for traditional Chinese medicine and food, respectively [35]. The root tubers of *P. lobata* show a higher isoflavone content, especially puerarin, which is called kudzu in the Chinese Pharmacopoeia. However, the root tubers of *P. thomsonii* show a higher starch content but a lower isoflavone content, and therefore it is called starch kudzu, which is generally used for food [36]. The genome sequence, transcriptome and metabolite analyses reported in the study might help understand the biosynthesis of these natural products.

Previously, Shang reported the first high-quality chromosome-scale genome of *P. thomsonii* [13]. The genome size was ~1.37 GB, with a contig N50 of 593.7 kb. The genome structural annotation resulted in 869.33 Mb repeat regions (62.7% of the genome) and 45,270 protein-coding genes. A total of 572 genes that were upregulated in the puerarin biosynthesis pathway were identified, and 235 candidate genes were further enriched by transcriptome data [13]. As another wild species of Pueraria, the genome size of *P. montana* was ~978.59 Mb, with contig N50 of 80.18 Mb. A comparative genomics analysis showed that the genome size of *P. montana* was smaller than that of *P. thomsonii* because of fewer repetitive sequences and duplicated genes [14]. Compared with the previous two species of pueraria, the genome of *P. lobata* was smaller and had fewer repetitive sequences and duplicated genes: the genome size of *P. lobata* was only ~939 Mb. The genome reported in *P. lobata* shows that the repetitive sequences accounted for 63.50% of the *P. lobata* genome, and a total of 33,171 coding genes were predicted, of which 97.34% could predict the function. A total of 224 genes related to flavonoid metabolism were enriched in this study, including 40 genes related to the isoflavone synthesis. *P. lobata* and *P. montana* were both wild species of pueraria, with less artificial cultivation and intervention. However, because of the low content of medicinal ingredients, *P. montana* was not used as traditional Chinese medicine [3,36]. *P. thomsonii* was more edible than medicinal in evolution, with faster growth speed and stronger edible roots, due to the artificial introduction and the change in the cultivation environment. The growth of *P. lobata* was slower, and it had higher isoflavone content. These findings may be related to genome duplication and changes in evolution.

Although *P. lobata* and *P. thomsonii* both were recorded in the Chinese Pharmacopoeia as having high medicinal value, modern studies have confirmed large differences between the two varieties in material basis and efficacy [37]. Shang’s study showed that *P. lobata* had higher amounts of syringaresinol-4′O-glucoside and disinapoyl glucoside, and *P. thomsonii* had higher amounts of glycycoumarin and 2-hydroxyadenosine. It was suggested that the specific compounds found in a particular variety could be used to differentiate the Pueraria varieties [36]. We detected 223 flavonoids in *P. lobata*, including 42 isoflavones. Besides the daidzein and puerarin commonly found in Pueraria plants, more isoflavones were also detected, such as daidzein-7-O-(2′’-benzoyl) rhamnoside, daidzein-7-O-Glucoside-4′-O-Apioside, genistein-7-O-(6′’-malonyl) glucoside, licoisoflavone B, calycosin-7-O-glucoside and other substances. These substances not only enriched isoflavones in *P. lobata*, but also increased the content of its medicinal active ingredients. These new active substances, which were different from those in *P. thomsonii*, might be related to the expansion of the glycosidase gene in the *P. lobata* genome.

As an isoflavonoid, puerarin is biosynthesized via the phenylpropanoid pathway by the hydroxylation of liquiritigenin at the C-2 position to yield its isoflavonoid skeleton. However, the reaction step for C-glucosylation in puerarin biosynthesis remains an enigma [4]. Based on the labeling studies in *P. lobata* roots, the chalcone substrate (isoliquiritigenin), but not the isoflavone substrate (daidzein), was purported to be an intermediate in the pathway to puerarin [38]. Wang’s study revealed that PlUGT43 possessed an activity for the C-glucosylation of daidzein to puerarin, and it showed activity with the isoflavones daidzein and genistein, but displayed no activity towards other potential acceptors, including flavonoids [4]. In this study, six unique PlUGT43 homologous genes were retrieved from the genome of *P. lobata*, and no 2-hydroxyisoflavanone 8-C-glucoside was found in the metabolites. This also confirmed that puerarin was synthesized mainly from the glycation of daidzein (Figure 1).

Besides the influence of structural genes in the metabolic pathway on puerarin synthesis, TFs are also important switches for regulating isoflavone synthesis. However, reports about the role of TFs in the regulation of puerarin synthesis are rare [4,39]. The TF regulation of the flavonoid pathway has been extensively studied in many plant species, such as *Z. mays*, *A. thaliana*, *M. domestica* and so on [40,41,42]. Among these, TFs of MYB, bHLH and WD40 function individually or collaborate as an MBW complex to control multiple enzymatic steps in the flavonoid pathway [43]. The biosynthesis of flavonoids or isoflavonoids has been extensively studied in model plants, but not in non-model plants due to the lack of genomic and genetic information. Shen found that the transcription levels of *PlMYB1*, *PlHLH3-4* and *PlWD40-1* genes were closely correlated with isoflavonoid accumulation profiles in different tissues and cell cultures of kudzu [39]. The over-expression of *PlMYB1* in *A. thaliana* significantly increased the accumulation of anthocyanins in leaves and pro-anthocyanidins in seeds by activating *AtDFR*, *AtANR* and *AtANS* genes [39]. The combined analysis of the genome and transcriptome of *P. thomsonii* indicated that 41 *PlbHLHs* showed root-specific expression patterns, and seven of them exhibited upregulated expression after MeJA treatment. Using qRT-PCR validation, five puerarin synthesis-related genes had a similar expression pattern as the seven *PlbHLH* genes in response to MeJA within 24 h [13]. In this study, 68 TFs related to isoflavone and puerarin synthesis were obtained in the *P. lobata* genome, including two *bHLH*, six *MYB* and four *WRKY*. The data suggested that these key TFs might be involved in the expression regulation of the structural genes of the puerarin synthesis pathway.

## 5. Conclusions

This study reported a chromosome-level genome assembly for *P. lobata* and its characteristics. *P. lobata* has a smaller genome size and fewer annotated genes compared with the cultivated variety *P. thomsonii*. The genome size is ~934 Mb, with a contig N50 of 29.51 Mbp. Approximately 97.82% of the assembled sequences are represented by 11 pseudochromosomes. We identified that the repetitive sequences accounted for 63.50% in the genome, and a total of 33,171 coding genes were predicted, of which 97.34% could predict the function. Compared with other leguminous plants, the genome of *P. lobata* has undergone significant contraction and expansion. The genome evolution analysis revealed that *P. lobata* was closely related to *G. max* and underwent two ancient WGD events. The multi-omics analysis revealed that not only did *4CL*, *CHR*, *CHI* and other genes participate in the synthesis of puerarin and daidzein, but also *WRKY*, *bHLH*, *MYB* and other TFs participated in the expression regulation of structural genes. In particular, six unique *PlUGT43* homologous genes were retrieved from the genome of *P. lobata*, and no 2-hydroxyisoflavanone 8-C-glucoside was found in the metabolites. We speculated that puerarin was synthesized mainly from the glycation of daidzein, and homologous *PlUGT* further catalyzed the conversion of puerarin into puerarin-4′-o-glucoside. This study developed the important multi-omics data for exploiting and improving *P. lobata* as an economic crop for edible traditional Chinese medicine.

## Figures and Tables

**Table 1:** Statistics of cluster number and length of single chromosome.

Chromosome ID	Cluster Number	Sequeues Length
Chr1	18	81,633,236
Chr2	10	106,209,211
Chr3	17	102,321,577
Chr4	11	103,164,207
Chr5	10	105,908,545
Chr6	14	69,390,138
Chr7	8	64,621,794
Chr8	8	81,655,469
Chr9	11	72,387,295
Chr10	14	64,242,115
Chr11	19	67,181,021

## Data Availability

This Whole Genome Shotgun project and Transcription project of *Pueraria lobata* (wild.) Ohwi has been deposited in DDBJ/ENA/GenBank and SRA under the umbrella of BioProject Accession PRJNA872914. The genome accession described in this paper is SRR21196526. The relevant data of metabolome have been uploaded to the GSA database, with the login number of OMIX002296-01, and the login numbers of transcriptome data are OMIX002296-02 and OMIX002296-03.

## Supplementary Materials

The following supporting information can be downloaded at: https://www.mdpi.com/article/10.3390/biom12121731/s1, Figure S1. According to the HIC data obtained by sequencing, mount the assembled contings/scaffolds sequence to the near chromosome level with Allhic software, and then manually correct it according to the intensity of chromosome interaction with juicebox software to finally obtain the genome at the chromosome level. Figure S2. Analysis of gene expansion and contraction enrichment in *P. lobata*. Figure S3. Positively selected genes enrichment analysis in genome of *P. lobata*. Figure S4. Collinearity analysis of chromosomes between *P. lobata* and other leguminous plants. Figure S5. Evolutionary analysis of *PlUGT43* gene family in puerarin synthesis pathway. Table S1 *P. lobata* sample collection information. Table S2 *P. lobata* genome assembly was accomplished using analysis tools. Table S3 Plant species used for gene family clustering in each species. Table S4 Statistics of genomic sequencing data of *P. lobata*. Table S5 Genome mount rate. Table S7 Genome assembly results. Table S6 CEGMA evaluation results of *P. lobata* genome. Table S7 Statistics of read coverage of *P. lobata* genome. Table S8 SNP statistics of *P. lobata* genome. Table S9 Repeat sequence statistical results. Table S10 Statistics of repeated sequence classification results. Table S11 Basic statistical results of gene structure prediction. Table S12 Basic statistical results of gene structure of near source species. Table S13 Statistical results of gene function annotation in different databases. Table S14 Statistical results of noncoding RNA in *P. lobata* genome. Table S15 Gene annotation related to puerarin metabolism in this study. Table S16 Metabolomic analysis of isoflavone content in two *P. lobata* sample.

## References

[1] Heo H.S., Han G.E., Won J., Cho Y., Woo H., Lee J.H. (2019). *Pueraria montana* var. lobata root extract inhibits photoaging on skin through Nrf2 pathway. J. Microbiol. Biotechnol..

[2] Adolfo L.M., Rao X., Dixon R.A. (2022). Identification of Pueraria spp. through DNA barcoding and comparative transcriptomics. BMC Plant Biol..

[3] Wang Y. (2020). Study on the Detection and Efficacy of Isoflavones Components in Pueraria in Different Regions in China. Master’s Thesis.

[4] Wang X., Li C., Zhou C., Li J., Zhang Y. (2017). Molecular characterization of the C-glucosylation for puerarin biosynthesis in *Pueraria lobata*. Plant J..

[5] Li Z., Li C., Li J., Zhang Y. (2014). Molecular cloning and functional characterization of two divergent 4-coumarate: Coenzyme A ligases from Kudzu (*Pueraria lobata*). Biol. Pharm. Bull..

[6] Liu J., Shi Y., Lee Y. (2019). Applications of *Pueraria lobata* in treating diabetics and reducing alcohol drinking. Chin. Herb. Med..

[7] Li J., Li C., Gou J., Zhang Y. (2016). Molecular cloning and functional characterization of a novel isoflavone 3′-O-methyltransferase from *Pueraria lobata*. Front. Plant Sci..

[8] Han R., Takahashi H., Nakamura M., Yoshimoto N., Suzuki H., Shibata D., Yamazaki M., Saito K. (2015). Transcriptomic landscape of *Pueraria lobata* demonstrates potential for phytochemical study. Front. Plant Sci..

[9] Pan Z., Yan G., Wang L., Xu X., Pan L. (2011). Effects of puerarin on blood pressure, blood lipid and renal structure of cold-induced hypertensive mice. Chin. J. Appl. Physiol..

[10] Wang C., Xu N., Cui S. (2021). Comparative transcriptome analysis of roots, stems, and leaves of *Pueraria lobata* (Willd.) Ohwi: Identification of genes involved in isoflavonoid biosynthesis. PeerJ.

[11] Vogt T. (2010). Phenylpropanoid biosynthesis. Mol. Plant.

[12] Meng Q., Huang J., Liang Y., Teng Y., Wu Y. (2020). Comparison of polysaccharides and isoflavones in *Pueraria thomsonii* and *Pueraria lobata* from different producing areas of Guangxi. Food Res. Dev..

[13] Shang X., Yi X., Xiao L., Zhang Y., Huang D., Xia Z., Ou K., Ming R., Zeng W., Wu D. (2022). Chromosomal-level genome and multi-omics dataset of *Pueraria lobata* var. thomsonii provide new insights into legume family and the isoflavone and puerarin biosynthesis pathways. Hortic. Res..

[14] Mo C., Wu Z., Shang X., Shi P., Wei M., Wang H., Xiao L., Cao S., Lu L., Zeng W. (2022). Chromosome-level and graphic genomes provide insights into metabolism of bioactive metabolites and cold-adaption of *Pueraria lobata* var. montana. DNA Res..

[15] He M., Yao Y., Li Y., Yang M., Li Y., Wu B., Yu D. (2019). Comprehensive transcriptome analysis reveals genes potentially involved in isoflavone biosynthesis in *Pueraria thomsonii* Benth. PLoS ONE.

[16] Rhoads A., Au K.F. (2015). PacBio sequencing and its applications. Genom. Proteom. Bioinform..

[17] Belaghzal H., Dekker J., Gibcus J.H. (2017). Hi-C 2.0: An optimized Hi-C procedure for high-resolution genome-wide mapping of chromosome conformation. Methods.

[18] Vasimuddin M., Misra S., Li H., Aluru S. Efficient architecture-aware acceleration of BWA-MEM for multicore systems. Proceedings of the 2019 IEEE International Parallel and Distributed Processing Symposium (IPDPS).

[19] Simão F.A., Waterhouse R.M., Ioannidis P., Kriventseva E.V., Zdobnov E.M. (2015). BUSCO: Assessing genome assembly and annotation completeness with single-copy orthologs. Bioinformatics.

[20] Bickhart D.M., Rosen B.D., Koren S., Sayre B.L., Hastie A.R., Chan S., Lee J., Lam E.T., Liachko I., Sullivan S.T. (2017). Single-molecule sequencing and chromatin conformation capture enable de novo reference assembly of the domestic goat genome. Nat. Genet..

[21] Rhie A., Walenz B.P., Koren S., Phillippy A.M. (2020). Merqury: Reference-free quality and phasing assessment for genome assemblies. bioRxiv.

[22] Camacho C., Coulouris G., Avagyan V., Ma N., Papadopoulos J., Bealer K., Madden T.L. (2009). BLAST+: Architecture and applications. BMC Bioinform..

[23] Yu X., Zheng H., Wang J., Wang W., Su B. (2006). Detecting lineage-specific adaptive evolution of brain-expressed genes in human using rhesus macaque as outgroup. Genomics.

[24] Li L., Stoeckert C.J., Roos D.S. (2003). OrthoMCL: Identification of ortholog groups for eukaryotic genomes. Genome Res..

[25] Edgar R.C. (2004). MUSCLE: Multiple sequence alignment with high accuracy and high throughput. Nucleic Acids Res..

[26] Stamatakis A. (2014). RAxML version 8: A tool for phylogenetic analysis and post-analysis of large phylogenies. Bioinformatics.

[27] Yang Z. (2007). PAML 4: Phylogenetic analysis by maximum likelihood. Mol. Biol. Evol..

[28] Hedges S.B., Dudley J., Kumar S. (2006). TimeTree: A public knowledge-base of divergence times among organisms. Bioinformatics.

[29] De Bie T., Cristianini N., Demuth J.P., Hahn M.W. (2006). CAFE: A computational tool for the study of gene family evolution. Bioinformatics.

[30] Wang Y., Tang H., DeBarry J.D., Tan X., Li J., Wang X., Lee T., Jin H., Marler B., Guo H. (2012). MCScanX: A toolkit for detection and evolutionary analysis of gene synteny and collinearity. Nucleic Acids Res..

[31] Cheng H., Zha S., Luo Y., Li L., Wang S., Wu S., Cheng S., Li L. (2022). JAZ1-3 and MYC2-1 synergistically regulate the transformation from completely mixed flower buds to female flower buds in *Castanea mollisima*. Int. J. Mol. Sci..

[32] Chen C., Chen H., Zhang Y., Thomas H.R., Xia R. (2020). TBtools: An integrative toolkit developed for interactive analyses of big biological data. Mol. Plant.

[33] Huang X., Huang X., Guo L., He L., Xiao D., Zhan J., Wang A., Liang R. (2022). Comparative transcriptome analysis provides insights into the resistance in Pueraria [*Pueraria lobata* (Willd.) Ohwi] in response to pseudo-rust disease. Int. J. Mol. Sci..

[34] Wang Q., Wang Y., Zhang W., Li K., Luo X., Cui Y. (2021). Puerarin from *Pueraria lobata* alleviates the symptoms of irritable bowel syndrome-diarrhea. Food Funct..

[35] Xie L. (2021). A Study on the Characteristics of Pharmacognosy of Three Varieties of Pueraria montana (Lour.) Merr.

[36] Shang X., Huang D., Wang Y., Xiao L., Ming R., Zeng W., Cao S., Lu L., Wu Z., Yan H. (2021). Identification of nutritional ingredients and medicinal components of *Pueraria lobata* and Its varieties using UPLC-MS/MS-based metabolomics. Molecules.

[37] Wong K.H., Razmovski-Naumovski V., Li K.M., Li G.Q., Chan K. (2015). Comparing morphological, chemical and anti-diabetic characteristics of *Puerariae Lobatae* Radix and *Puerariae Thomsonii* Radix. J. Ethnopharmacol..

[38] Inoue T., Fujita M. (1977). Biosynthesis of Puerarin in Pueraria Root. Chem. Pharm. Bull..

[39] Shen G., Wu R., Xia Y., Pang Y. (2021). Identification of transcription factor genes and functional characterization of PlMYB1 from *Pueraria lobata*. Front. Plant Sci..

[40] Espley R.V., Hellens R.P., Putterill J., Stevenson D.E., Kutty A.S., Allan A.C. (2007). Red colouration in apple fruit is due to the activity of the MYB transcription factor, MdMYB10. Plant J..

[41] Carey C.C., Strahle J.T., Selinger D.A., Chandler V.L. (2004). Mutations in the pale aleurone color1 regulatory gene of the *Zea mays* anthocyanin pathway have distinct phenotypes relative to the functionally similar TRANSPARENT TESTA GLABRA1 gene in *Arabidopsis thaliana*. Plant Cell.

[42] Wada T., Kunihiro A., Tominaga W.R. (2014). Arabidopsis CAPRICE (MYB) and GLABRA3 (bHLH) control tomato (*Solanum lycopersicum*) anthocyanin biosynthesis. PLoS ONE.

[43] Xu W., Grain D., Bobet S., Le Gourrierec J., Thévenin J., Kelemen Z., Lepiniec L., Dubos C. (2014). Complexity and robustness of the flavonoid transcriptional regulatory network revealed by comprehensive analyses of MYB–b HLH–WDR complexes and their targets in *Arabidopsis* seed. New Phytol..

