# Supplementary material for: Chromosome-Level Genome Assembly and Multi-Omics Dataset Provide Insights into Isoflavone and Puerarin Biosynthesis in Pueraria lobata (Wild.) Ohwi
Source: Biomolecules. 2022 Nov 22;12(12):1731. doi: 10.3390/biom12121731 (PMC9775041; doi:10.3390/biom12121731)
Supplement: Supplementary file 1 [file biomolecules-12-01731-s001.zip › biomolecules-1977562 - supplementary.pdf]

biomolecules

Supplementary Materials

Chromosome-Level Genome Assembly and Multi-Omics Dataset Provide Insights into Isoflavone and Puerarin Biosynthesis in *Pueraria lobata* (Wild.) Ohwi

Hua Cheng [1,2,3], Xiaohua Huang [3], Shuai Wu [1,2], Shiyan Wang [1,2], Shen Rao [1,2], Li Li [1,2], Shuiyuan Cheng [1,2] and Linling Li [1,2,3,*]

[1] School of Modern Industry for Selenium Science and Engineering, Wuhan Polytechnic University, Wuhan 430048, China
[2] National R&D Center for Se-Rich Agricultural Products Processing, Wuhan Polytechnic University, Wuhan 430023, China
[3] College of Biology and Agricultural Resources, Huanggang Normal University, Huanggang 438000, China
* Correspondence: 12622@whpu.edu.cn; Tel.: +86-173-7156-9920

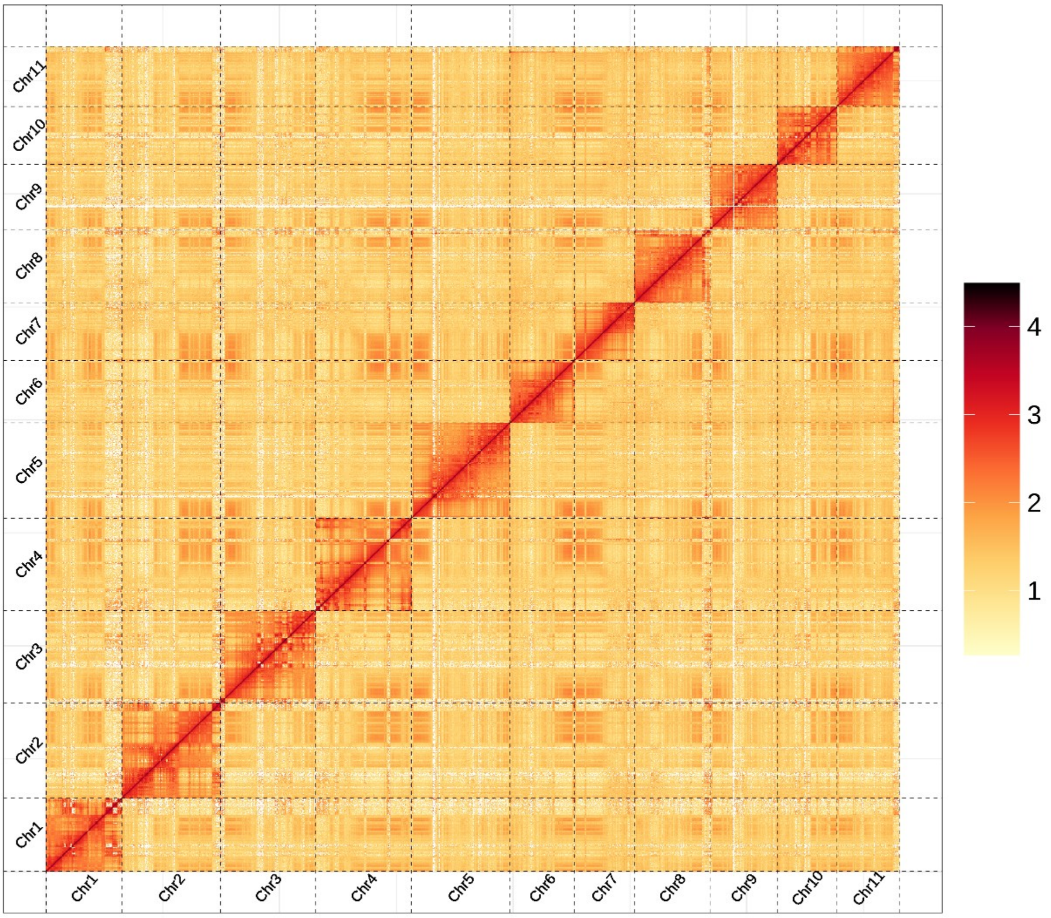

Figure S1. According to the HIC data obtained by sequencing, mount the assembled contings/scaffolds sequence to the near chromosome level with Allhic software, and then manually correct it according to the intensity of chromosome interaction with juicebox software to finally obtain the genome at the chromosome level.

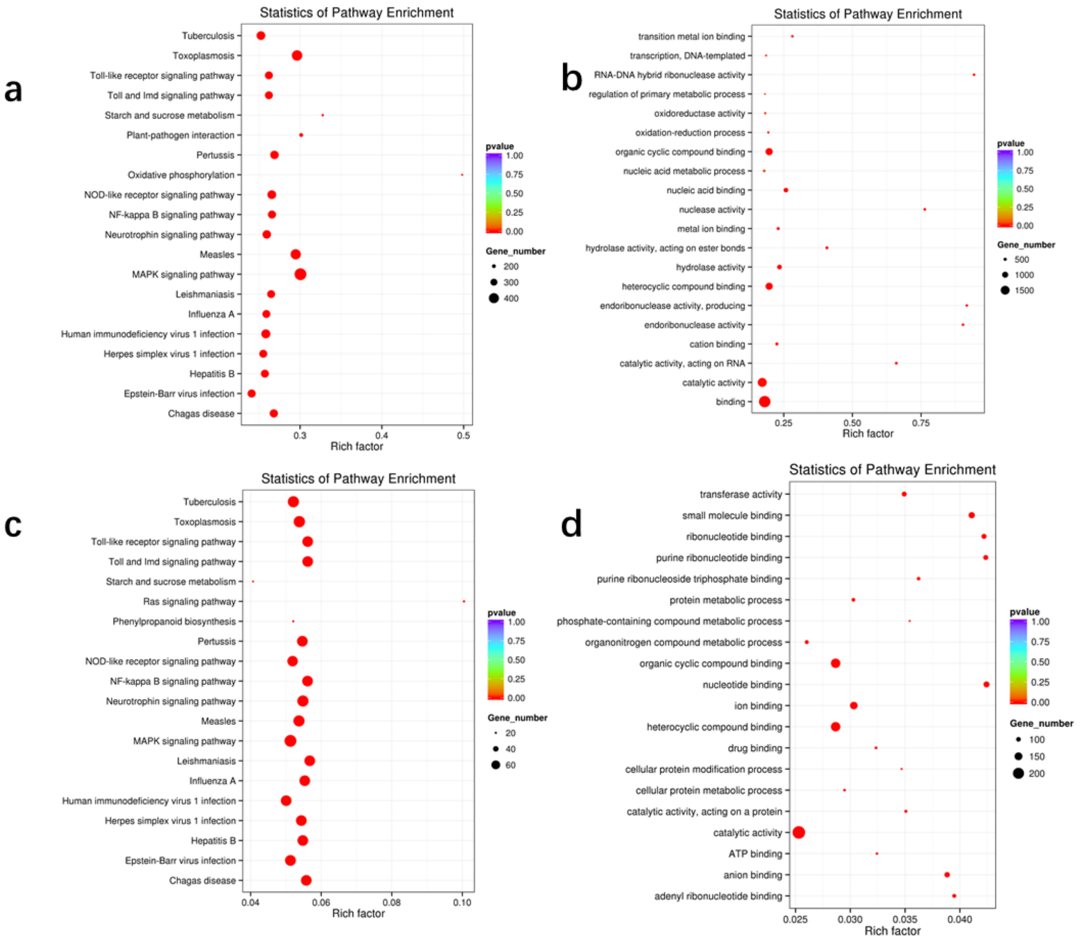

Figure S2. Analysis of gene expansion and contraction enrichment in *P. lobata*. (**a**) KEGG enrichment map of *P. lobata* expansion gene; (**b**) Go enrichment map of *P. lobata* expansion gene; (**c**) KEGG enrichment map of *P. lobata* contraction gene; (**d**) Go enrichment map of *P. lobata* contraction gene.

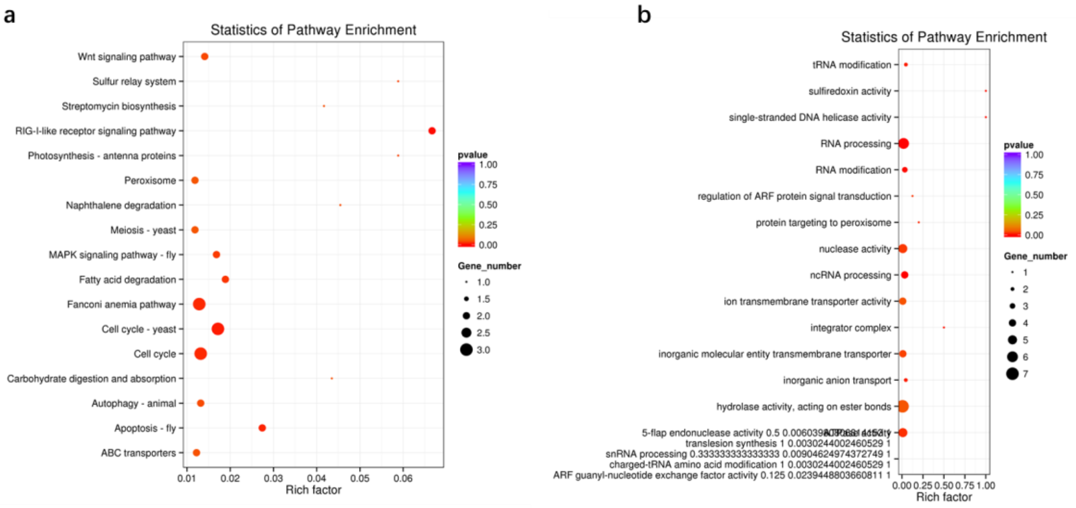

Figure S3. Positively selected genes enrichment analysis in genome of *P. lobata*. (**a**) KEGG enrichment map; (**b**) GO KEGG enrichment map.

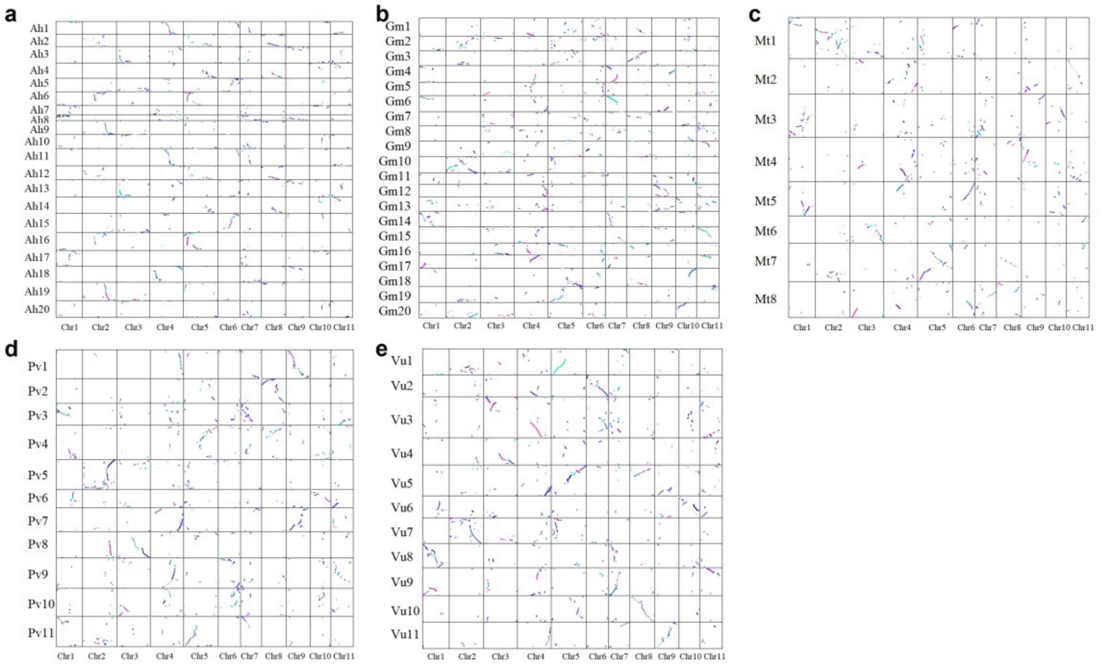

Figure S4. Collinearity analysis of chromosomes between *P. lobata* and other leguminous plants. The abscissa represents the chromosome number of *P. lobata*, and the abscissa represents the chromosome number of five comparative leguminous plants. (**a**). *P. lobata* and *A. hypogaea* (Ah1 to Ah20); (**b**). *P. lobata* and *G. max* (Gm1 to Gm20); (**c**). *P. lobata* and *M.truncatula* (Mt1 to Mt8); (**d**). *P. lobata* and *P. vulgaris* (Pv1 to Pv11); (**e**). *P. lobata* and *V. unguiculata* (Vu1 to Vu11).

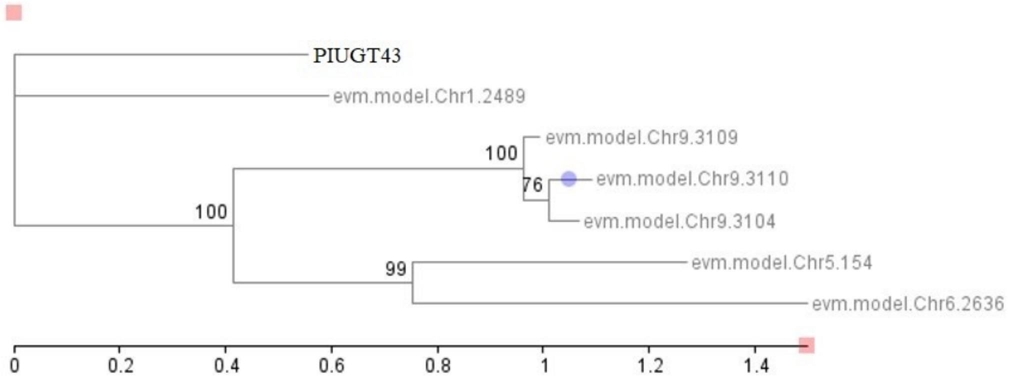

Figure S5. Evolutionary analysis of *PlUGT43* gene family in puerarin synthesis pathway.

Table S1. *P. lobata* sample collection information.

Sample name	Name in the paper	Place	Purpose	Total flavone content	Tissue	Time
PlobLT13	A	Luotian, China	DNA and RNA extraction, Gene annotation		Young leaves	2021.04
PlobLT13	A	Luotian, China	Gene annotation		Stem	2021.10
PlobLT13	A	Luotian, China	Gene annotation		Flower	2021.07
PlobLT13	A	Luotian, China	Gene annotation		Seeds	2021.10
PlobLT13	A	Luotian, China	Gene annotation, Transcriptome and Metabolome analysis	7.13 %	Root	2021.10
Plob53	B	Wuhan, China	Transcriptome and Metabolome analysis	7.96 %	Roots	2021.10
Plob17	C	Wuhan, China	Transcriptome and Metabolome analysis	11.57 %	Roots	2021.10
Plob19	D	Wuhan, China	Transcriptome and Metabolome analysis	9.34 %	Roots	2021.10
Plob25	E	Wuhan, China	Transcriptome and Metabolome analysis	10.21 %	Roots	2021.10

Table S2. *P. lobata* genome assembly was accomplished using analysis tools.

Analysis process	Software name	Version
Assemble	Hifiasm	0.16.1
	pilon	1.22
	Racon	V1.3.1
	Arrow	Smartlink8.0
Polish	Nextpolish	1.3.1
	Bionano Solve	V3.5
	LACHESIS	201701
Auxiliary assembly	ALLHIC	0.9.8
	BUSCO	V5.2.1
	CGEMA	V2.5
	LTR_retriever	V2.9.0
	BWA	0.7.8
Evaluation	Samtools	0.1.19

Table S3. Plant species used for gene family clustering in each species.

Code name	Latin name of species	Abbreviation	Number of selected genes
Plob	*Pueraria_lobata*	*P. lobata*	33171
Atha	*Arabidopsis_thaliana*	*A. thaliana*	27310
Gmax	*Glycine_max*	*G. max*	53593
Mtru	*Medicago_truncatula*	*M. truncatula*	50385
Adur	*Arachis_duranensis*	*A. duranensis*	33952
AhyTA	*Arachis_hypogaea*	*A. hypogaea*	31241
Pvul	*Phaseolus_vulgaris*	*P. vulgaris*	32390
Ccaj	*Cajanus_cajan*	*C. cajan*	28619
Cari	*Cicer_arietinum*	*C. arietinum*	28269
Vrad	*Vigna_radiata*	*V. radiata*	42188
Tpra	*Trifolium_pratense*	*T. pratense*	39247
Lalb	*Lupinus_albus*	*L. albus*	37995
Vung	*Vigna_unguiculata*	*V. unguiculata*	29434
Vang	*Vigna_angularis*	*V. angularis*	33860

Table S4. Statistics of genomic sequencing data of *P. lobata.*

Libraries	Insert size	Total data (G)	Read length (bp)	Sequence coverage (%)
Illumina reads	350 bp	57.99	150	62.49
PacBio reads	--	26.62	--	28.69
Total	--	84.61	--	91.18

Table S5. Genome mount rate.

Class	Scaffold Number	Total Length
place	11	918,714,608
unplace	234	20,499,398
total	245	939,214,006
Rate	97.82%	

Table S6. Genome assembly results.

Title	Total_length	Total_number	Max_length	N50_length	N50_number	N90_length	N90_number
Contig	939,201,106	374	60,107,759	29,506,772	11	5,213,946	36
Scaffold	939,201,106	374	60,107,759	29,506,772	11	5,213,946	36

Table S7. CEGMA evaluation results of *P. lobata* genome.

	Complete		Complete + Partial	
Species	Prots	Completeness	Prots	Completeness
P. lobata	235	94.76 %	240	96.77 %

Table S8. Statistics of read coverage of *P. lobata* genome.

Category	Comparison parameters	Percentage
Reads	Mapping rate	98.79 %
Genome	Average sequencing depth	48.30 %

Coverage	99.97 %
Coverage at least 4X	99.90 %
Coverage at least 10X	99.60 %
Coverage at least 20X	95.36 %

Table S9. SNP statistics of *P. lobata* genome.

Category	Number	Percentage
All SNP	4,261,874	0.468853%
Heterozygosis SNP	4,261,248	0.468784%
Homology SNP	626	6.9e-05%

Table S10. Repeat sequence statistical results.

Trf	128,676,572	13.70
Repeat masker	582,141,440	61.98
Protein mask	74,334,641	7.91
Total	596,376,034	63.50

Total is the result obtained by the above methods and the non-redundant result after removing the overlap between them.

Table S11. Statistics of repeated sequence classification results.

	Denovo+Repbase		TE Proteins		Combined TEs	
	**Length(bp)**	**Percentage**	**Length(bp)**	**Percentage**	**Length(bp)**	**Percentage**
DNA	55,570,876	5.92	1,325,320	0.14	56,438,405	6.01
LINE	20,393,616	2.17	12,168,686	1.30	28,026,905	2.98
SINE	771,583	0.08	0	0	771,583	0.08
LTR	465,000,075	49.51	60,844,456	6.48	467,707,528	49.80
Unknown	62,853,315	6.69	0	0	62,853,315	6.69
Total	582,141,440	61.98	74,334,641	7.91	586,253,309	62.42

Note: The statistical results do not include TRF identification results. Denovo + RepBase is the library predicted by RepeatModeler, RepeatScout, Piler and LTR_Finder software, and the transposon elements obtained by using RepeatMasker software to annotate the genome after integrating the nucleic acid Library of RepBase with the software of uclust according to the principle of 80-80-80.the transposon elements obtained by annotating the genome with RepeatMasker software; TE proteins annotate the transposon elements obtained from the genome by RepeatProteinmask based on the RepBase protein library. Combined TEs is the result of integrating the above two methods and eliminating redundancy. Unknown means that the repeat sequence cannot be classified by RepeatMasker. Total is the result of the above classification, and the non-redundant result after removing the overlapping parts between them.

Table S12. Basic statistical results of gene structure prediction.

	Gene set	Number	Average transcript length(bp)	Average CDS length(bp)	Average exons per gene	Average exon length(bp)	Average intron length(bp)
De novo	Augustus	45,087	2,705.40	975.24	4.19	232.85	542.66
	GlimmerHMM	38,084	10,201.76	662.62	3.23	205.36	4,284.11
	SNAP	18,707	40,864.76	543.77	4.35	125.09	12,047.16
	Geneid	60,670	4,948.13	740.39	3.98	185.92	1,410.89
	Genscan	40,496	11,336.61	1,070.26	5.31	201.54	2,381.80
Homo-log	*Vigna angularis*	27,356	3,246.96	1,111.69	4.58	242.60	596.03
	Arachis duranensis	27,237	3,197.53	1,121.69	4.60	243.70	576.17

RNAseq	*Medicago truncatula*	30,958	2,966.55	1,030.45	4.31	238.87	584.27
	Arabidopsis thaliana	24,987	3,063.83	1,096.43	4.45	246.48	570.52
	Glycine max	35,325	2,850.52	977.99	4.17	234.60	590.94
	PASA	29,579	3,477.35	1,206.29	4.96	243.05	573.03
	Transcripts	50,847	14,925.77	1,769.11	5.75	307.67	2,769.84
	EVM	45,893	3,173.15	978.69	4.25	230.50	676.06
	Pasa update*	45,832	3,140.30	985.08	4.25	231.93	663.68
	Final set*	33,171	3,785.50	1,170.28	4.98	234.81	656.43

Note: *Include UTR region, others do not.

Table S13. Basic statistical results of gene structure of near source species.

Species	Number	Average transcript length(bp)	Average CDS length(bp)	Average exons per gene	Average exon length(bp)	Average intron length(bp)
Plob	33,171	3,785.50	1,170.28	4.98	234.81	656.43
Gmax	55,651	3,145.79	1,172.88	5.06	231.75	485.82
Mtru	50,385	2,336.61	991.33	4.07	243.76	438.66
Vang	33,860	2,876.28	1,117.03	4.49	248.75	504.00
Adur	52,826	3,497.07	1,296.02	5.53	234.36	485.87
Atha	27,310	1,887.74	1,229.90	5.16	238.41	158.18

Table S14. Statistical results of gene function annotation in different databases.

Database	Number	Percent (%)
Total	33,171	-
Swissprot	25,416	76.62
Nr	31,736	95.67

KEGG	25,462	76.76
InterPro	31,348	94.50
GO	19,508	58.81
Pfam	25,055	75.53
Annotated	32,290	97.34
Unannotated	881	2.66

Table S15. Statistical results of noncoding RNA in *P. lobata* genome.

Type		Copy number	Average length(bp)	Total length(bp)	Percentage in genome
miRNA		1,843	127.21	234,440	0.024961
tRNA		2,417	75.79	183,178	0.019503
rRNA	rRNA	5,886	372.08	2,190,065	0.23
	18S	887	1,684.31	1,493,987	0.16
	28S	3,234	140.01	452,805	0.048211
	5.8S	826	168.08	138,835	0.014782
	5S	939	111.22	104,438	0.011120
snRNA	snRNA	746	119.99	89,512	0.009531
	CD-box	482	103.04	49,665	0.005288
	HACA-box	75	142.43	10,682	0.001137
	splicing	184	153.40	28,226	0.003005
	scaRNA	5	187.80	939	0.000100
	Unknown	0	0	0	0

Table S16. Metabolomic analysis of isoflavone content in two *P. lobata* sample.

Index	Compounds	A1	A2	A3	C1	C2	C3
MWSHY0147	Daidzein	1.40E+06	1.56E+06	1.34E+06	3.52E+06	2.60E+06	3.45E+06
Zmdp004370	6''-O-Acetyldaidzin	3.98E+05	3.77E+05	3.90E+05	5.41E+05	5.63E+05	5.72E+05
pme1587	Daidzein-7-O-glucoside (Daidzin)	2.44E+06	2.79E+06	2.62E+06	3.41E+06	3.66E+06	3.42E+06
mws0063	Puerarin	1.88E+05	1.52E+05	1.81E+05	2.53E+05	2.73E+05	2.85E+05
MWSHY0180	Glycitin	6.36E+06	5.83E+06	8.07E+06	9.65E+06	7.90E+06	8.69E+06
Zmdp004305	Daidzein-7-O-(2''-benzoyl)rhamnoside	5.05E+06	5.27E+06	5.45E+06	6.00E+06	6.10E+06	6.27E+06
Zmdp003677	Daidzein-7-O-Glucoside-4'-O-Apioside	5.16E+06	5.46E+06	5.17E+06	4.20E+06	4.40E+06	4.51E+06
Zmdp004112	Genistein-7-O-(6''-malonyl)glucoside	1.54E+07	1.66E+07	1.55E+07	1.67E+07	1.62E+07	1.63E+07
mws0908	Glycitein	1.38E+05	1.36E+05	1.40E+05	2.85E+05	4.64E+05	4.40E+05
mws0037	2,7,4'- trihydroxyisoflavone	3.44E+05	2.85E+05	3.13E+05	5.18E+05	5.30E+05	5.41E+05
pmp000362	Glisoflavone	6.74E+04	6.72E+04	6.85E+04	4.99E+04	2.91E+04	3.48E+04
pmp000195	6''-O-Malonylglycitin	1.66E+07	1.54E+07	1.69E+07	1.75E+07	1.69E+07	1.71E+07
Lmgp003335	4',5-Dihydroxyisoflavone-7-O-galactoside	1.91E+07	1.81E+07	1.99E+07	2.88E+07	2.88E+07	2.86E+07
pmp000193	6''-O-Malonyldaidzin	1.56E+06	1.70E+06	1.84E+06	1.76E+06	1.99E+06	1.94E+06
pmp000194	6''-O-Malonylgenistin	9.34E+06	7.94E+06	8.72E+06	1.18E+07	1.11E+07	1.12E+07
Lmhn005110	Aracarpene 1	1.91E+05	1.77E+05	1.79E+05	2.14E+05	1.85E+05	1.92E+05
Lmhn004976	Aracarpene 2	1.91E+05	1.77E+05	1.79E+05	2.14E+05	1.85E+05	1.92E+05
mws0912	Liquiritigenin	1.98E+05	2.04E+05	1.96E+05	4.66E+05	5.21E+05	4.89E+05
Lmmp004504	2'-Hydroxygenistein	1.13E+05	1.10E+05	1.11E+05	1.12E+05	1.16E+05	2.10E+04
MWSHY0063	Isoliquiritigenin	1.06E+06	1.07E+06	1.14E+06	2.11E+06	1.92E+06	1.86E+06
mws0895	Genistein-7-O-Glucoside	1.29E+06	1.46E+06	7.77E+05	1.88E+06	2.04E+06	2.05E+06
pmp000363	Gancaonin N	2.45E+05	2.21E+05	2.56E+05	2.45E+05	2.03E+05	1.95E+05
pmn001390	Licoisoflavone B	1.22E+04	1.19E+04	1.18E+04	6.56E+03	7.05E+03	6.53E+03
MWSHY0111	Calycosin-7-O-glucoside	1.03E+07	1.02E+07	9.68E+06	1.45E+07	1.35E+07	1.43E+07
pme3233	Calycosin	1.31E+05	2.73E+05	1.41E+05	2.71E+05	2.72E+05	2.74E+05

Zmdp003228	Daidzein-7-O-apiosyl(1→6)glucoside	5.12E+06	5.44E+06	5.02E+06	4.32E+06	4.11E+06	3.59E+06
Zmdp005767	Formononetin-7-O-(6''-Malonyl)glucoside	2.06E+06	1.67E+06	1.68E+06	3.03E+06	2.70E+06	4.82E+06
MWSHY0179	Formononetin-7-O-glucoside (Ononin)	1.04E+06	9.44E+05	9.79E+05	1.30E+06	1.31E+06	1.41E+06
Lmdn009383	Glyceollin III	4.14E+04	3.99E+04	4.53E+04	2.22E+04	1.98E+04	1.85E+04
pmp000191	6''-O-Acetylgenistin	6.58E+04	6.04E+04	6.60E+04	1.20E+05	1.07E+05	1.17E+05
Lmdn006025	2-Hydroxy-2,3-dihydrogenistein	2.21E+05	2.34E+05	2.38E+05	8.11E+05	5.60E+05	5.34E+05
Lmdn007639	3,9-Dihydroxypterocarpan	2.90E+06	2.72E+06	1.95E+06	2.20E+06	2.18E+06	3.29E+06
Lmhp003217	2'-Hydoxy-5-methoxyGenistein-O-rhamnosyl-glucoside	1.48E+05	1.17E+05	1.39E+05	1.26E+05	1.51E+05	1.24E+05
pmp000414	Puerarin-4'-o-glucoside	2.71E+05	2.68E+05	2.72E+05	4.83E+05	4.47E+05	4.65E+05
Lssp210437	3'-O-methylorobol	4.37E+04	4.62E+04	4.22E+04	4.58E+04	3.71E+04	4.71E+04
Lmdp003110	2,6,7,4'-Tetrahydroxyisoflavanone	1.23E+05	1.17E+05	1.12E+05	1.92E+05	1.98E+05	2.28E+05
pmp000192	6''-O-Acetylglycitin	1.85E+04	2.85E+04	1.78E+04	3.12E+04	1.89E+04	2.50E+04
pmp000395	Glycyroside	1.34E+05	1.37E+05	1.51E+05	7.72E+04	8.12E+04	7.45E+04
pmp000417	Daidzein-4'-O-glucoside	1.10E+06	9.97E+05	1.20E+06	1.49E+06	1.76E+06	1.66E+06
Lmdp003994	6,4'-Dimethoxyisoflavone-7-O-glucoside (Wistin)	1.74E+06	1.10E+06	1.31E+06	1.23E+06	1.15E+06	1.05E+06
Wmkn002777	7-Hydroxy-3''-methoxy-isoflavone-7-primeveroside	3.57E+04	3.70E+04	2.93E+04	2.10E+04	1.20E+04	1.61E+04

Table S17. Gene annotation related to puerarin metabolism in this study.

Gene_ID	A1	A2	A3	C1	C2	C3	NR
GLYMA_01G091400	0	0	0	0	0	0	chalcone synthase 6-like
GLYMA_02G257700	0.4	0	0	0	0	0	leucoanthocyanidin dioxygenase-like
GLYMA_02G309300	41.7	43.72	38.13	43.35	49.83	35.93	phenylalanine ammonia-lyase class 3 isoform X1
GLYMA_03G181600	78.46	101.37	94.16	220.07	252.13	234.76	phenylalanine ammonia-lyase 1
GLYMA_03G181700	0	0	0	0	0	0.27	phenylalanine ammonia-lyase 1

GLYMA_04G222400	24.85	29.49	53.71	52.95	34.7	29.07	chalcone isomerase 4B
GLYMA_01G232400	2233.23	2369.02	2070.92	1455.7	1332.78	1432.78	4-coumarate CoA ligase (*4CL*)
GLYMA_08G109500	10.26	18.96	8.28	35.29	36.21	32.04	chalcone synthase 1
GLYMA_09G211500	1.24	0.9	1.37	2.36	2.95	0.81	isoflavone reductase homolog
GLYMA_10G058200	70.7	56.17	61.86	80.77	81.38	103.87	phenylalanine ammonia-lyase
GLYMA_10G209800	0	0.25	0	0	0	0	phenylalanine ammonia-lyase 2-like isoform X2
GLYMA_11G097900	0	0	0	0	0	0	type III polyketide synthase B-like
GLYMA_13G145000	19.97	4.47	8.2	7.55	13.57	23.04	phenylalanine ammonia-lyase class 2
GLYMA_18G285800	138.41	114.06	159.9	132.69	158.4	136.95	chalcone reductase (CHR) like
GLYMA_19G105100	0.43	1.41	0	1.64	0.44	2.52	chalcone synthase J-like
GLYMA_19G182300	4.33	5.62	11.07	38.39	31.2	163.45	phenylalanine ammonia-lyase 1
GLYMA_20G180800	5.34	5.6	8.16	8.01	5.44	5.62	phenylalanine ammonia-lyase 2
GLYMA_20G241500	0	0.59	0	0	0	0	chalcone--flavonone isomerase 1A
GLYMA_02G174800	17.74	3.06	1.72	2.96	2.86	0.3	transcription factor bHLH48-like
GLYMA_04G220500	4.74	1.59	2.62	2.6	2.79	0.53	8-C-glucosyltransferase
GLYMA_05G241000	13.68	5.97	4.23	10.41	7.81	4.82	glucosyltransferase (GT) gene family
GLYMA_10G180100	11.04	1.51	2.03	0.44	2.34	0.45	cinnamate-4-hydroxylase
GLYMA_05G178200	2.33	6.73	9.2	3.17	3.06	3.1	2-hydroxyisof lavanonesynthase
GLYMA_06G160500	1.03	1.12	11.92	4.4	1.05	10.05	MYB transcription factor MYB56
GLYMA_08G118200	0	0	3.25	0.93	0.5	0.96	WRKY48 protein
GLYMA_09G254800	5.95	3.03	0.44	2.64	2.43	0	PREDICTED: WRKY transcription factor 22-like
GLYMA_11G215800	3.16	6.91	13.98	0	6.45	5.75	2-hydroxyisoflavanone dehydratase.
